# Factors associated with grade ≥2 acute radiation dermatitis after adjuvant radiotherapy in breast cancer patients with immediate breast reconstruction: a single-center study using penalized regression

**DOI:** 10.3389/fonc.2026.1903847

**Published:** 2026-08-18

**Authors:** Yadong Liu, Xuejuan Duan, Cunkai Wang, Yibo Li, Yaying Wu, Zixuan He, Peihong Lian, Na Li, Xianbo Zhang, Jing Zhao

**Affiliations:** 1 Department of Oncology, Hebei General Hospital, Shijiazhuang, China; 2 Department of Radiation Oncology, Fourth Hospital of Hebei Medical University, Shijiazhuang, China; 3 Geriatric Gastroenterology Department, Hebei General Hospital, Shijiazhuang, China; 4 Hebei Medical University, Shijiazhuang, China

**Keywords:** breast cancer, breast reconstruction, penalized regression, post-mastectomy radiotherapy, radiation dermatitis, regional nodal irradiation

## Abstract

**Background:**

Immediate breast reconstruction is increasingly performed in women with breast cancer, yet many such patients also require post-mastectomy radiotherapy (PMRT), during which acute radiation dermatitis (RD) is common and may compromise reconstruction outcomes and quality of life. Risk factors for RD have been characterized mainly in non-reconstructed or breast-conserving populations, and data specific to immediately-reconstructed patients are scarce.

**Methods:**

We retrospectively analyzed 61 women with immediate reconstruction (implant/expander or autologous tissue) who completed adjuvant radiotherapy. All patients received intensity-modulated radiotherapy (IMRT) delivered in 25 fractions with a routine 0.5-cm chest-wall bolus; the prescribed chest-wall/breast target dose was ≥50 Gy (EQD2 50 Gy) in 57 of 61 patients (93%) and slightly lower (<50 Gy) in the remaining 4. Acute RD was graded with the Radiation Therapy Oncology Group (RTOG) scale and dichotomized at grade ≥2. Univariable comparisons used the Mann -Whitney U and Fisher exact tests. Because of the small sample, imbalanced outcome and quasi-separation, Firth penalized-likelihood logistic regression with profile penalized-likelihood 95% confidence intervals (CIs) was used as the primary method. A parsimonious model (age plus supraclavicular irradiation) was pre-specified from clinical priors and univariable P<0.10, and evaluated by likelihood-ratio test, area under the receiver-operating-characteristic curve (AUC) and 1000-fold bootstrap optimism correction.

**Results:**

Grade ≥2 RD occurred in 72.1% of patients (44/61). In the multivariable model, older age (odds ratio [OR] 2.30 per 10 years, 95% CI 1.11 -5.87; P=0.023) and supraclavicular irradiation (OR 6.40, 95% CI 1.37 -37.37; P=0.018) were associated with grade ≥2 RD; the event rate was 76.9% (40/52) with versus 44.4% (4/9) without supraclavicular irradiation. The model likelihood-ratio χ^2^ was 16.30 (P=0.0003); the apparent AUC was 0.719 and the optimism-corrected AUC 0.705. Findings were consistent in an exploratory sensitivity analysis additionally adjusting for tumour laterality.

**Conclusion:**

In conclusion, grade ≥2 acute RD was common after adjuvant radiotherapy in immediately-reconstructed patients, and older age and supraclavicular (regional nodal) irradiation were associated with the outcome. These hypothesis-generating findings support intensified skin-protective strategies for high-risk patients and warrant validation in larger, multicenter cohorts.

## 1 Introduction

Breast reconstruction has become an integral component of the surgical management of breast cancer, improving body image and psychosocial functioning, and an increasing proportion of patients now choose reconstruction (1). However, for patients with an indication for post-mastectomy radiotherapy (PMRT), balancing local tumor control against reconstruction outcomes remains a practical challenge. Radiotherapy and reconstruction interact: radiotherapy increases reconstruction-related complications and the risk of capsular contracture (2, 3), whereas the altered chest-wall geometry produced by an implant/expander or autologous flap may in turn affect skin dose and acute reactions.

Acute radiation dermatitis is one of the most common adverse effects of breast radiotherapy, affecting the majority of patients (up to ~80%) (4); it ranges from erythema to dry and moist desquamation and, in severe cases, leads to treatment interruption, infection, and impaired quality of life. In reconstructed patients, it may even jeopardize the survival and cosmetic outcome of the reconstructed tissue, making skin protection particularly important in this group. Previous studies of RD risk factors have focused largely on breast-conserving or non-reconstructed populations receiving whole-breast/chest-wall irradiation, implicating body mass index, diabetes, smoking, breast volume, conventional fractionation, boost, and bolus, whereas the role of regional nodal irradiation remains controversial (5–7). Data on RD risk factors specific to the growing population undergoing PMRT after immediate breast reconstruction, however, remain relatively scarce.

Given the relative rarity of this population and the realistic constraints on single-center sample size, conventional maximum-likelihood logistic regression is prone to bias—or may fail to converge—in small samples with an imbalanced outcome or quasi-separation (8, 9). We therefore analyzed, on the basis of a faithful description of real-world data, the factors associated with grade ≥2 acute RD in patients undergoing adjuvant radiotherapy after immediate breast reconstruction, using robust methods including Firth penalized-likelihood regression, with the aim of providing clues for identifying high-risk patients and guiding skin protection.

## 2 Materials and methods

### 2.1 Study design and patients

This was a single-center retrospective cohort study. Consecutive eligible patients were treated at a single institution between February 2024 and April 2025. Eligible patients were women with pathologically confirmed breast cancer who underwent mastectomy with immediate breast reconstruction (implant/tissue expander or autologous tissue flap) and received adjuvant radiotherapy with complete dosimetric and skin-reaction records. A total of 61 patients were included, with no missing values for any variable. All patients underwent immediate reconstruction.

### 2.2 Data collection and variable definitions

Demographic and clinical variables included age, height, weight, body mass index (BMI), body surface area, educational level, economic status, diabetes mellitus, endocrine therapy, targeted therapy, chemotherapy, tumor laterality, pathological type, and TNM stage. All patients were treated with intensity-modulated radiotherapy (IMRT) delivered in 25 fractions with a 0.5-cm tissue-equivalent bolus applied to the chest wall throughout the treatment course. The prescribed dose to the chest-wall/breast target was ≥50 Gy (EQD2–50 Gy; BED10–60 Gy) in 57 patients (93%) and <50 Gy in the remaining 4 patients (7%), the latter reflecting individualized dose de-escalation at the treating team’s discretion. Because the treatment technique, fractionation schedule, and bolus were uniform across the cohort, they were not entered as variables. The radiotherapy variable “chest-wall/breast dose ≥50 Gy” therefore denotes the prescribed target dose (≥50 vs <50 Gy) and whether each regional target was irradiated: supraclavicular region (CTVsc), posterior/level-II supraclavicular region (CTVscnd), internal mammary region (CTVim), internal mammary nodes (CTVimnd), and axilla (CTVax); the number of irradiated nodal regions was derived accordingly. Detailed skin dosimetry (e.g., skin Dmax/Dmean or irradiated skin volumes such as V105%/V107%) was not retrievable from the records. The outcome was acute radiation dermatitis, graded with the Radiation Therapy Oncology Group (RTOG) acute radiation morbidity scale (10). Acute RD was recorded as the overall (highest) RTOG grade for the treated field; the anatomical subsite of the most severe reaction was not separately documented. Using the clinically relevant “moderate-to-severe” threshold, grade ≥2 (marked erythema/patchy moist desquamation or worse) was defined as the event and compared with grade 1 (mild). Acute radiation dermatitis was independently assessed by two board-certified radiation oncologists, strictly according to the RTOG acute radiation morbidity scoring criteria, at each weekly on-treatment visit and again at approximately 2 weeks after completion of radiotherapy. The highest grade recorded over the entire course (including the 2-week posttreatment assessment) was defined as the final outcome. When the two assessors disagreed, the grade was resolved by discussion; where consensus could not be reached, a third senior radiation oncologist adjudicated the final grade. As this was a retrospective study based on routine clinical documentation, formal blinding of assessors and formal interobserver reliability testing (e.g., kappa statistics) were not performed.

### 2.3 Statistical analysis

Continuous variables are summarized as median (interquartile range, IQR) and categorical variables as counts (percentages). For univariable comparisons, continuous variables were analyzed with the Mann–Whitney U test and categorical variables with the Fisher exact test. Given the small sample (n=61), the imbalanced outcome (grade ≥2 in 72.1%; only 17 controls) and the highly skewed distribution of several exposures with quasi-separation, conventional maximum-likelihood logistic regression may yield biased or non-convergent estimates. The primary analysis therefore used Firth penalized-likelihood logistic regression (8, 9), which reduces small-sample bias through a Jeffreys-prior penalty on the likelihood and yields finite estimates even under separation. This method is primarily designed to mitigate small-sample and sparse-event estimation bias, rather than to address model overfitting. Odds ratios (ORs) and 95% CIs were obtained by the profile penalized-likelihood method, and P values were based on penalized likelihood-ratio tests. To avoid overfitting, the multivariable model was prespecified—on the basis of clinical plausibility and univariable P<0.10—to include only two variables, age (continuous) and supraclavicular irradiation. Model performance was assessed by the overall likelihood-ratio test, the AUC and the Brier score; optimism was estimated from 1,000 bootstrap resamples and used to internally correct the AUC. A sensitivity analysis additionally included tumor laterality. Tests were two-sided, with P<0.05 considered significant. Analyses were performed in Python 3 (statsmodels, scipy, scikit-learn).

## 3 Results

### 3.1 Patient characteristics and incidence of radiation dermatitis

**Figure 1:**
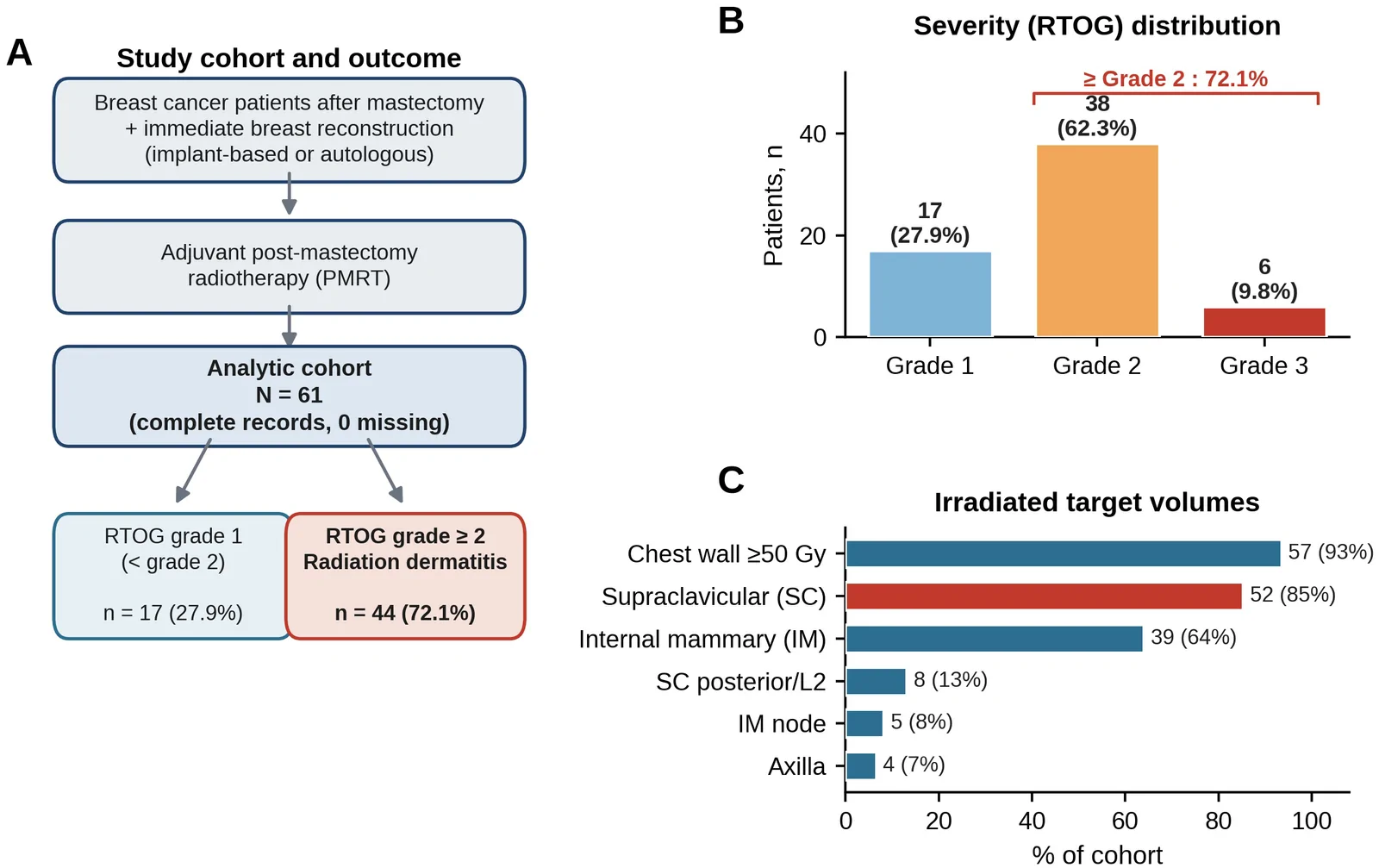
Study cohort and outcome overview. **(A)** Patient selection and grouping; **(B)** distribution of acute radiation dermatitis by RTOG grade, with grade ≥2 accounting for 72.1%; **(C)** frequency of irradiated target volumes. SC, supraclavicular; IM, internal mammary.

The 61 patients had a median age of 40 years (IQR 37–49) and a median BMI of 24.6 kg/m². Most tumors were invasive breast carcinoma, and the TNM stage was predominantly II (57%) and III (31%). Regarding radiotherapy, 93% (57/61) received ≥50 Gy to the chest-wall/breast target (i.e., the prescribed target dose), and regional irradiation was frequent: supraclavicular region in 85% (52/61) and internal mammary region in 64% (39/61). Acute RD was grade 1 in 17 patients (27.9%), grade 2 in 38 (62.3%), and grade 3 in 6 (9.8%); grade ≥2 thus occurred in 44 patients, an incidence of 72.1% (**Figure 1**). The distribution of baseline variables between the grade 1 and grade ≥2 groups and the univariable comparisons are shown in **Table 1**.

**Table 1:** Baseline, clinical and radiotherapy characteristics and their association with grade ≥2 acute radiation dermatitis (n=61).

Variable	Overall (n=61)	RTOG grade 1 (n=17)	RTOG grade ≥2 (n=44)	P value
Age, years [M (IQR)]	40.0 (37.0–49.0)	38.0 (36.0–41.0)	42.5 (37.0–49.2)	0.075
Body mass index, kg/m² [M (IQR)]	24.6 (21.6–27.2)	24.6 (22.5–26.2)	24.6 (21.4–27.9)	0.729
Body surface area, m² [M (IQR)]	1.6 (1.5–1.8)	1.6 (1.5–1.7)	1.7 (1.6–1.8)	0.201
No. of irradiated nodal regions [M (IQR)]	2.0 (1.0–2.0)	2.0 (0.0–2.0)	2.0 (1.0–2.0)	0.340
Educational level (≥ high school)	32 (52)	7 (41)	25 (57)	0.392
Economic status (low income)	50 (82)	14 (82)	36 (82)	1.000
Diabetes mellitus (yes)	4 (7)	1 (6)	3 (7)	1.000
Endocrine therapy (yes)	39 (64)	12 (71)	27 (61)	0.565
Targeted therapy (yes)	4 (7)	1 (6)	3 (7)	1.000
Chemotherapy (yes)	59 (97)	15 (88)	44 (100)	0.074
Right-sided tumor	30 (49)	6 (35)	24 (55)	0.255
TNM stage				0.159
Stage 0–I	5 (8)	3 (18)	2 (5)	
Stage II	35 (57)	7 (41)	28 (64)	
Stage III	19 (31)	7 (41)	12 (27)	
Stage IV	2 (3)	0 (0)	2 (5)	
Chest-wall/breast dose ≥50 Gy	57 (93)	15 (88)	42 (95)	0.308
Supraclavicular irradiation (yes)	52 (85)	12 (71)	40 (91)	0.100
Posterior/level-II supraclavicular (yes)	8 (13)	2 (12)	6 (14)	1.000
Internal mammary irradiation (yes)	39 (64)	9 (53)	30 (68)	0.373
Internal mammary nodes (yes)	5 (8)	2 (12)	3 (7)	0.612
Axillary irradiation (yes)	4 (7)	1 (6)	3 (7)	1.000

Continuous variables are median (IQR); categorical variables are n (%). P values are from the Mann–Whitney U or Fisher exact test. Chest-wall/breast dose ≥50 Gy’ denotes the prescribed target dose. All patients were treated in 25 fractions.

Most variables did not differ significantly between groups (**Table 1**), suggesting limited marginal effects of any single factor in this reconstructed population; age (median 42.5 vs 38 years; P = 0.075) and supraclavicular irradiation (91% vs 71%; P = 0.100) showed trend-level differences.

### 3.2 Univariable and multivariable Firth regression

**Figure 2:**
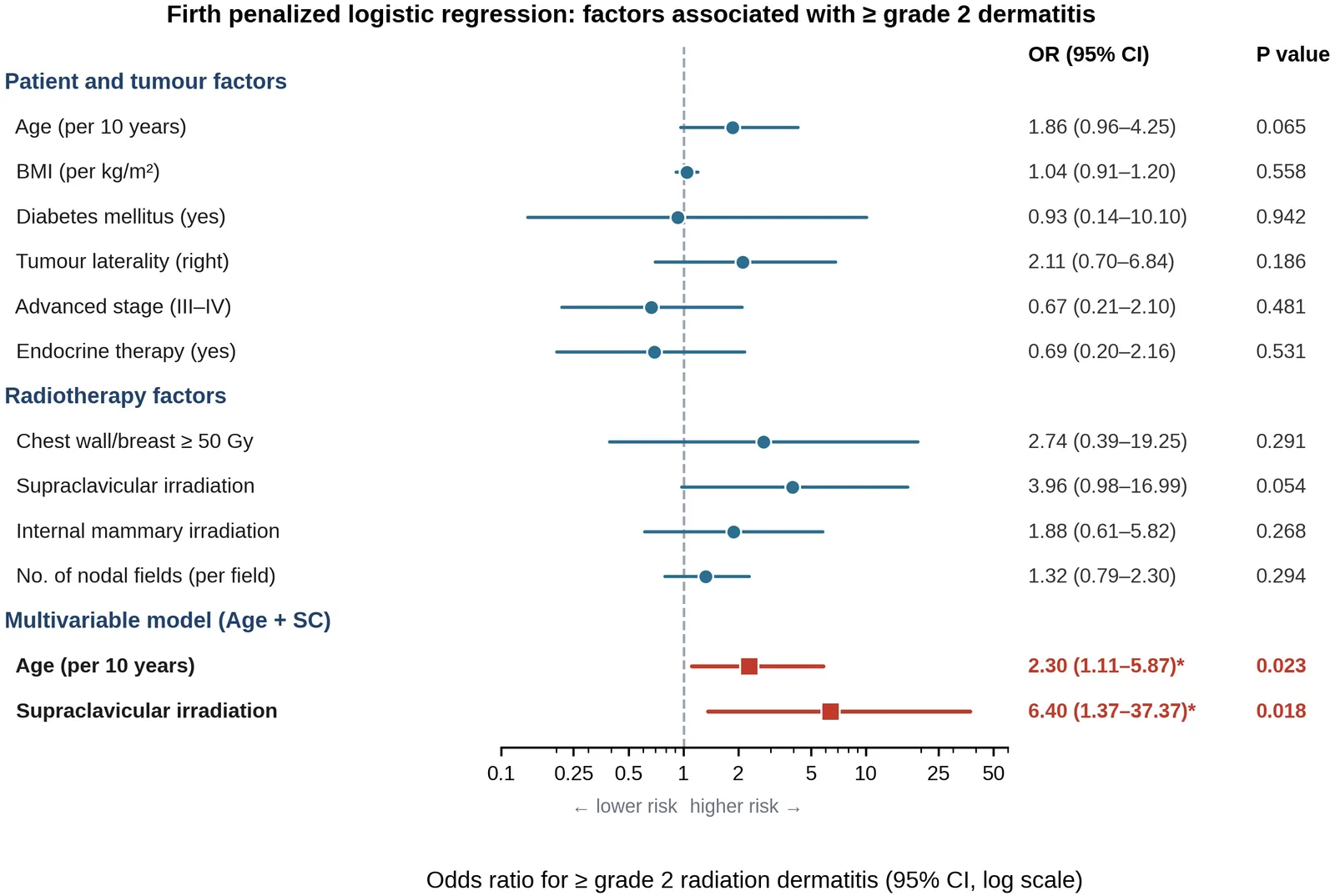
Forest plot of factors associated with grade ≥2 acute radiation dermatitis (Firth penalized regression). Upper rows, univariable analysis; lower red rows, the multivariable model (age + supraclavicular irradiation). The x-axis shows odds ratios with 95% CIs on a log scale; *P<0.05.

In univariable Firth regression, age (per 10 years: OR 1.86, 95% CI 0.96–4.25; P = 0.065) and supraclavicular irradiation (OR 3.96, 95% CI 0.98–16.99; P = 0.054) were borderline associated with grade ≥2 RD, whereas the remaining variables (BMI, diabetes, tumor laterality, TNM stage, endocrine therapy, chest-wall/breast dose, internal mammary irradiation, and number of irradiated regions) did not reach significance (**Table 2**, **Figure 2**). In the prespecified multivariable model, both variables were significant after mutual adjustment: each 10-year increase in age was associated with an OR of 2.30 (95% CI 1.11–5.87; P = 0.023; per year OR 1.09, 95% CI 1.01–1.19), and supraclavicular irradiation with an OR of 6.40 (95% CI 1.37–37.37; P = 0.018) relative to no supraclavicular irradiation. This pattern borderline univariable associations becoming significant after adjustment suggests mild mutual suppression/confounding, with each effect emerging more clearly once the other was accounted for. Given the small number of patients without supraclavicular irradiation (n=9) and the correspondingly wide confidence interval, this pattern should be interpreted with caution and regarded as exploratory rather than as evidence of definitive independent effects. The overall model likelihood-ratio χ² was 16.30 (df=2, P = 0.0003).

**Table 2:** Firth penalized-likelihood logistic regression for grade ≥2 acute radiation dermatitis.

Factor	OR (95% CI)	P value
Univariable analysis (Firth penalized regression)		
Age (per 10 years)	1.86 (0.96–4.25)	0.065
Body mass index (per 1 kg/m²)	1.04 (0.91–1.20)	0.558
Diabetes mellitus (yes vs no)	0.93 (0.14–10.10)	0.942
Right-sided tumor (vs left)	2.11 (0.70–6.84)	0.186
Advanced stage (III–IV vs 0–II)	0.67 (0.21–2.10)	0.481
Endocrine therapy (yes vs no)	0.69 (0.20–2.16)	0.531
Chest-wall/breast ≥50 Gy (vs <50)	2.74 (0.39–19.25)	0.291
Supraclavicular irradiation (yes vs no)	3.96 (0.98–16.99)	0.054
Internal mammary irradiation (yes vs no)	1.88 (0.61–5.82)	0.268
No. of irradiated nodal regions (per region)	1.32 (0.79–2.30)	0.294
Multivariable model (age + supraclavicular irradiation)		
Age (per 10 years)	2.30 (1.11–5.87)	0.023
Supraclavicular irradiation (yes vs no)	6.40 (1.37–37.37)	0.018

ORs and 95% CIs were estimated by the profile penalized-likelihood method. For the multivariable model, the overall likelihood-ratio χ² was 16.30 (P = 0.0003); the apparent AUC was 0.719, the optimism-corrected AUC 0.705 and the Brier score 0.167.

### 3.3 Key associations, joint effect, and model performance

**Figure 3:**
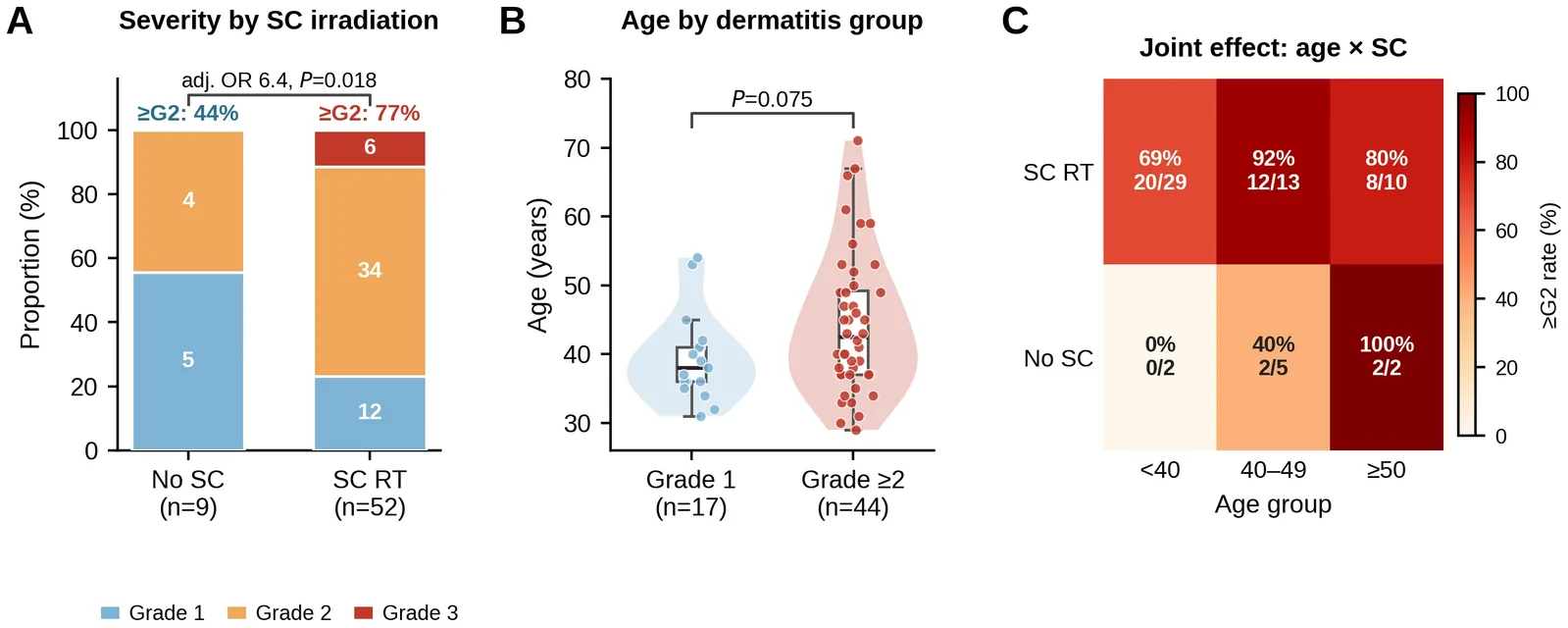
Key associations and joint effect. **(A)** RTOG-grade composition and grade ≥2 rate by supraclavicular irradiation; **(B)** age distribution by dermatitis group (violin + box + jitter); **(C)** heatmap of the grade ≥2 rate by age group × supraclavicular irradiation (cells show rate and events/total).

Among patients receiving supraclavicular irradiation, the incidence of grade ≥2 RD was 76.9% (40/52), markedly higher than the 44.4% (4/9) among those without it (**Figure 3A**). The grade ≥2 group was older overall than the grade 1 group (**Figure 3B**). Joint stratification by age and supraclavicular irradiation showed an additive trend (**Figure 3C**): among irradiated patients, the incidence rose with age (69% at <40 years, 92% at 40–49 years), suggesting that older patients who also receive regional irradiation are at particularly high risk.

**Figure 4:**
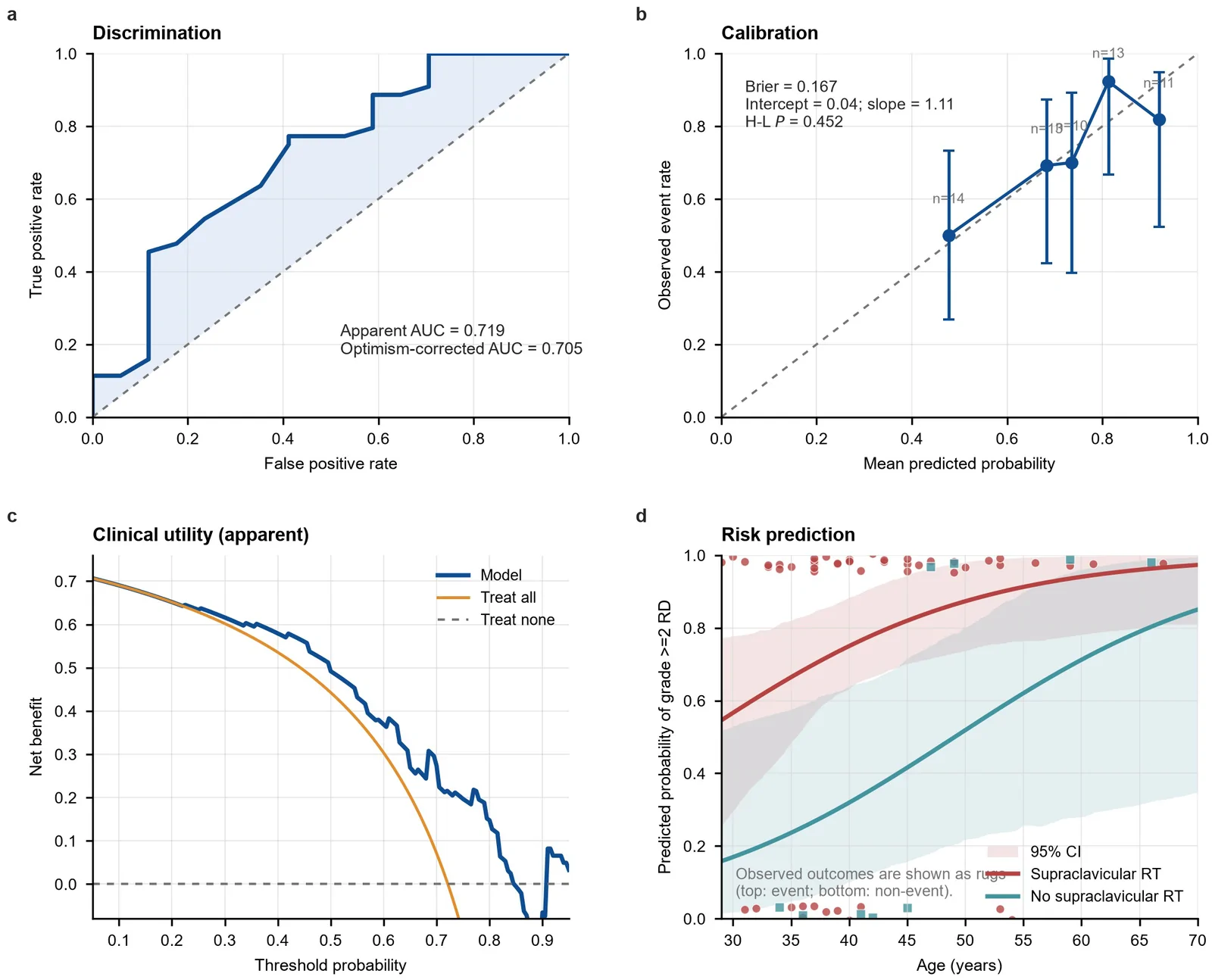
Internal validation and risk prediction of the multivariable model. **(A)** ROC curve/discrimination performance. The apparent AUC of the model is 0.719, and the bootstrap-corrected AUC is 0.705. This indicates that the model can moderately distinguish patients with grade ≥2 radiation dermatitis, yet it does not serve as a strong predictive model. **(B)** Calibration curve. The calibration points are generally close to the ideal diagonal line. The Brier score is 0.167, the calibration intercept is 0.04 (close to 0), and the calibration slope is 1.11 (close to 1); the Hosmer–Lemeshow test yields a P value of 0.452, suggesting no obvious poor model fit. **(C)** Decision curve analysis (DCA). Within the threshold probability range of approximately 0.225–0.95, the model outperforms both the “treat all” and “treat none” strategies. This suggests potential, exploratory clinical utility for flagging higher-risk patients (predicted risk ≳20–25%) for intensified skin care and monitoring; because discrimination and net benefit were assessed only by internal bootstrap optimism correction, external validation is required before any clinical use. **(D)** Risk prediction curve. The predicted risk of grade ≥2 radiation dermatitis (RD) rises with advancing age; patients receiving supraclavicular irradiation exhibit markedly higher risks at the same age. This aligns with the multivariate regression results: age and supraclavicular irradiation are major risk predictors.

The model built from age and supraclavicular irradiation had an apparent AUC of 0.719; the bootstrap-estimated optimism was only 0.014, giving an optimism-corrected AUC of 0.705 and a Brier score of 0.167, indicating moderate yet stable discrimination with low overfitting risk (**Figure 4**). According to the model, the predicted probability of grade ≥2 RD increased markedly with age among patients receiving supraclavicular irradiation (**Figure 4d**). Decision-curve analysis suggested potential net benefit across clinically relevant threshold probabilities, although external validation remains necessary before clinical implementation.

### 3.4 Sensitivity analysis

After additionally including tumor laterality in the multivariable model, age (OR 1.08 per year, 95% CI 1.01–1.18; P = 0.033) and supraclavicular irradiation (OR 6.83, 95% CI 1.44–41.37; P = 0.016) remained associated with the outcome, whereas a right-sided tumor did not (OR 2.14, 95% CI 0.65–7.75; P = 0.214), indicating that the principal findings were consistent across this model specification; this should be regarded as an internally supported, exploratory observation.

## 4 Discussion

In this study focused on the specific population undergoing adjuvant radiotherapy after immediate breast reconstruction, grade ≥2 acute RD was strikingly common (72.1%), and older age and supraclavicular (regional nodal) irradiation were identified as correlates; the resulting model showed moderate, internally stable discrimination.

The grade ≥2 incidence in our cohort was clearly higher than in several reports dominated by breast-conserving or mixed populations. For example, using the same RTOG scale, Nanthong et al. (11) (635 patients) reported a moderate-to-severe (grade ≥2) incidence of ~32%, and the real-world HYPOBREAST cohort (12) (Bruand et al., 1727 patients), which included patients receiving supraclavicular irradiation, reported 28.4%. We propose that the higher incidence observed here may be attributable to several factors. All patients underwent immediate reconstruction, in which an implant/expander or flap alters chest-wall geometry and displaces the skin to a more superficial position, thereby increasing skin dose through a bolus-like effect. Notably, a 0.5-cm tissue-equivalent bolus was applied to the chest wall in every patient; because a bolus raises the surface (skin) dose toward the prescription level, its routine use is a likely major contributor to the high incidence observed, consistent with a systematic review in which bolus markedly increased acute grade 3 dermatitis (9.6% vs 1.2%) (16). In addition, a high proportion of patients received regional irradiation—supraclavicular in 85% and internal mammary in 64%—which entailed larger treatment fields, and conventional fractionation with a prescribed dose of ≥50 Gy predominated, accounting for 93% of cases. These features together may have elevated the acute skin response. Importantly, grading scales and thresholds differ across studies, so direct comparisons of incidence should be made cautiously.

The association between supraclavicular irradiation and grade ≥2 RD is biologically plausible. Supraclavicular/regional irradiation substantially enlarges the treatment field, and this region has thin skin, often lies within skin folds, and is subject to field-junction and superficial high-dose effects, predisposing to more severe acute reactions. Because treatment technique, fractionation, and bolus were uniform across our cohort, this association is unlikely to be explained by differences in these factors; however, in the absence of skin dosimetry and of the anatomical site of the most severe reaction, we cannot determine whether it reflects larger field size, field-junction hot spots, or higher regional skin dose, and the finding should therefore be regarded as exploratory. Our finding is consistent with Parekh et al. (7), who identified regional nodal irradiation as an independent predictor of moist desquamation (OR 3.29) and reported the highest moist-desquamation rate among PMRT patients (24%); randomized trials such as MA.20 (Whelan et al. (13)) and EORTC 22922 (Poortmans et al. (14)) likewise show that adding regional (including internal mammary and supraclavicular) irradiation to whole-breast/chest-wall treatment increases related acute and late toxicity. Notably, the number of irradiated nodal regions per se was not significant (OR 1.32; P = 0.294), suggesting that risk relates more to involvement of the superficial supraclavicular region specifically than to the number of fields alone.

The association of older age with more severe RD also accords with prior evidence. Both Nanthong et al. and Xie et al. (15) (2023) reported age as a factor for acute RD. First, aging is accompanied by structural degeneration of the skin: epidermal atrophy, reduced and disorganized dermal collagen fibers, and atrophy of skin appendages, which collectively impair the skin barrier function and tolerance to radiation injury. Second, elderly patients have impaired tissue repair capacity: the proliferative activity of keratinocytes and fibroblasts declines with age, angiogenesis is attenuated, and the inflammatory repair response after radiation damage is dysregulated, leading to delayed recovery and higher likelihood of progressing to moist desquamation. Third, cellular senescence is associated with diminished DNA damage repair efficiency; senescent skin cells are more susceptible to radiation-induced cytotoxicity, resulting in more severe tissue injury at the same radiation dose. These age-related changes in skin structure, repair capacity, and intrinsic radiosensitivity jointly explain the higher risk of moderate-to-severe acute dermatitis in older patients. In our cohort, risk increased approximately 2.3-fold per 10 years, which is of clinical relevance.

Unlike most reports, we did not observe a significant association between BMI and grade ≥2 RD (OR 1.04; P = 0.558). A systematic review (Xie et al. (5); 38 studies, 15,623 patients) identified BMI ≥25 kg/m² as a robust risk factor (RR 1.11). The discrepancy may reflect our limited sample size, the all-reconstructed population (in whom chest-wall/skin dose is more strongly influenced by the reconstruction, possibly diluting the marginal effect of BMI), and differences in outcome thresholds and measurement. This difference warrants further examination in larger samples.

Methodologically, to address the small, imbalanced sample, we used Firth penalized-likelihood regression with profile-likelihood CIs. This approach is a recognized solution to separation and small-sample bias (8, 9) and is more robust than conventional maximum likelihood; together with a prespecified parsimonious model, exact tests, and bootstrap optimism correction, it helps reduce overfitting and false-positive risk in limited data. The very small estimated optimism (0.014) suggests adequate internal stability.

Clinically, older reconstructed patients who require supraclavicular irradiation should be regarded as a high-risk group for acute RD, warranting closer skin monitoring and prophylactic care during radiotherapy as well as active exploration of more favorable dose-distribution strategies such as hypofractionation, intensity-modulated/volumetric-modulated arc therapy, or proton therapy to reduce skin toxicity; hypofractionation has been shown to substantially lower the risk of acute RD in regionally irradiated patients (HYPOBREAST (12): OR 0.34; Nanthong et al. (11): adjusted OR 0.31). Because age and supraclavicular irradiation are baseline, largely non-modifiable characteristics, their principal value is to flag patients for closer monitoring and intensified prophylactic skin care rather than to indicate modification of the radiotherapy prescription itself. As the potentially modifiable technical factors were uniform in our cohort (IMRT, conventional fractionation and a routine 0.5-cm bolus), future studies should prospectively evaluate modifiable strategies—including hypofractionation and selective reduction or omission of bolus in patients without skin involvement (16) to reduce skin toxicity.

This study has several limitations. First, it was a single-center, retrospective study with a small, imbalanced sample (n=61), limited statistical power, and wide CIs for some ORs, so the findings are primarily hypothesis generating. Second, although treatment technique, fractionation, and bolus were uniform across the cohort (IMRT; 50 Gy in 25 fractions; routine 0.5-cm bolus)—which both limits generalizability and precludes evaluation of these factors as modifiable risk factors—we did not capture detailed skin dosimetry (e.g., skin Dmax/Dmean or irradiated skin volume), individual hot-spot or field-junction doses, the anatomical site of the most severe reaction, or smoking. In particular, reconstruction subtype (implant/expander vs autologous) was not recorded in a form suitable for adjustment, so we could not evaluate or adjust for its potential influence on skin dose and acute reactions; this should be addressed in future prospective studies. Third, RD was graded clinically without formal blinding or formal interobserver reliability (e.g., kappa) testing, introducing potential interobserver variability. Fourth, the multivariable significance partly reflects effects emerging after mutual adjustment and requires validation in larger samples. Future multicenter, prospective studies incorporating dosimetry and reconstruction subtype are needed to clarify causal and dose–response relationships.

## 5 Conclusion

Among breast cancer patients receiving adjuvant radiotherapy after immediate breast reconstruction, grade ≥2 acute radiation dermatitis was common, and older age and supraclavicular irradiation were associated with the outcome in this exploratory analysis and may help identify patients who warrant closer monitoring and preventive skin care. Intensified skin protection and optimized radiotherapy strategies are advisable for this high-risk group. Given the limited sample size, these findings require validation in larger prospective studies that incorporate skin dosimetry, the anatomical site of dermatitis, reconstruction subtype, and modifiable technical factors.

## Data Availability

The original contributions presented in the study are included in the article/supplementary material. Further inquiries can be directed to the corresponding author.
